# The Journey From Knee to Heart: A Case of Methicillin-Sensitive Staphylococcus aureus Infective Endocarditis Secondary to Septic Prepatellar Bursitis

**DOI:** 10.7759/cureus.36806

**Published:** 2023-03-28

**Authors:** Hafiza Qadeer, Amar Suwal, Ibiyemi O Oke

**Affiliations:** 1 Internal Medicine, Reading Hospital - Tower Health, Reading, USA

**Keywords:** mssa bacteremia, staphylococcus aureus, infective endocarditis, sepsis, pre-patellar bursitis

## Abstract

*Staphylococcus aureus* is a cause of life-threatening gram-positive bacteremia and the most common causative organism of septic bursitis. Although there are several case reports of infective endocarditis complicated with septic bursitis and other rheumatic complications, there are very few cases reports of septic bursitis leading to infective endocarditis (IE). We present a patient with a history of recurrent prepatellar bursitis requiring catheter drainage, who presented with sepsis and IE.

## Introduction

*Staphylococcus aureus *is a common cause of life-threatening gram-positive bacteremia and is associated with a substantial risk of infective endocarditis (IE), especially in patients with underlying predisposing factors. A transesophageal echocardiogram, in some cases, is important in the evaluation of patients with gram-positive bacteremia for prognostication and therapeutic reasons [1]. The prepatellar bursa is located superficially in the knee, where it is prone to trauma and subsequent infection due to thin overlying skin. The most common causative organism of septic bursitis is *S. aureus* in 80% of cases; less commonly reported organisms include coagulase-negative *Staphylococci*, *Enterococcus spp*, *Escherichia coli*, *Pseudomonas aeruginosa*, and anaerobes [2].

## Case presentation

**Figure 1:**
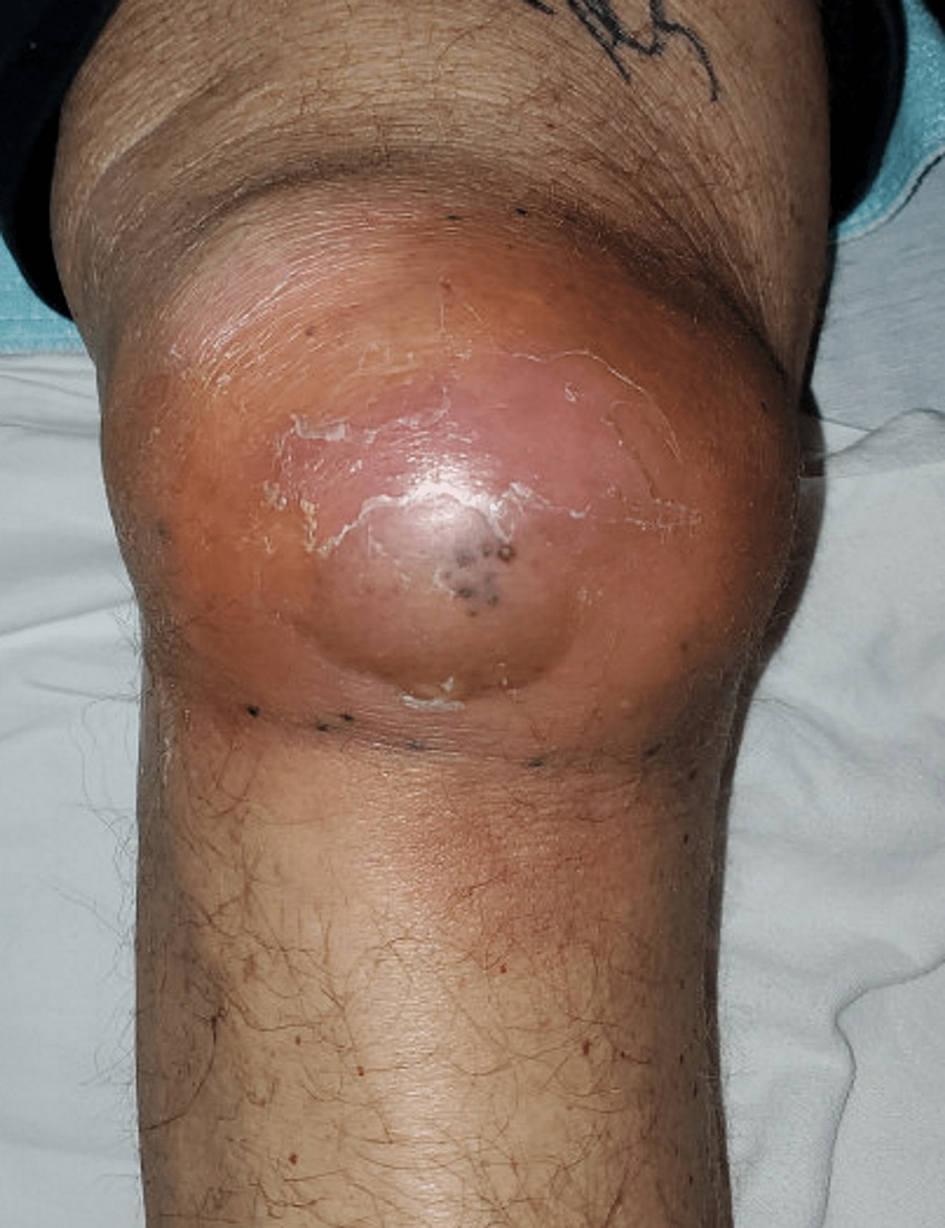
An image of the patient’s right knee at presentation showing swelling and overlying erythema

A 67-year-old with a past medical history of hypertension, moderate aortic stenosis, type 2 diabetes mellitus, hyperlipidemia, iron deficiency anemia, and a 10-year history of recurrent prepatellar bursitis with hemorrhagic effusion requiring catheter drainage (last drainage was done one week prior to this presentation), who worked as a bricklayer for more than 10 years, presented to the ER with two weeks history of worsening right knee swelling, pain, and difficulty with ambulation. There was associated redness of the skin overlying the swelling, loss of appetite, and chills but no fever, nausea, vomiting, night sweats, or malaise (Figure 1). No swelling was observed in other joints in the body.

**Figure 2:**
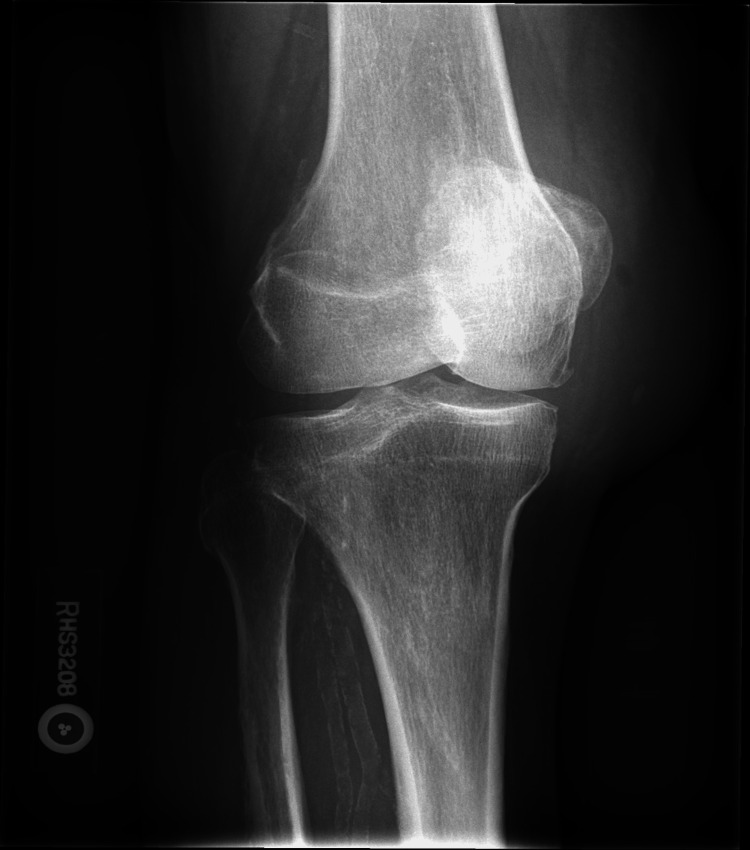
Anteroposterior view of patient’s right knee X-ray showing significant prepatellar swelling

In the ER, the patient had a temperature of 36.7 °C, heart rate of 63 bpm, respiratory rate of 16/min, and oxygen saturation (SPO2) of 99% on room air. There was marked swelling of the right knee with overlying erythema, differential warmth, and mild tenderness but no significant limitation of passive or active range of motion across the knee joint. He had a known 3/6 systolic ejection murmur in the aortic area but no physical stigmata of IE. His laboratory studies revealed a white cell count of 12.4*10E3/ul, hemoglobin 9.9g/dL, platelet 311, uric acid 7.0 (4.4mg/dL-7.6mg/dL), sedimentation rate 100 (0mm/hr-20mm/hr), C-reactive protein (CRP) 15.67 (<1.00mg/dL), creatinine 1.58, and an otherwise unremarkable basic metabolic panel. The X-ray of the right knee showed multicompartmental loss of joint space with osteophytosis and significant prepatellar soft tissue swelling (Figure 2).

The right knee joint aspirate was negative for crystals but grew methicillin-sensitive *S. aureus* (MSSA) and *Streptococcus mitis *group (Table 1).

**Table 1:** Synovial fluid analysis

Variable (unit)	Result	Normal value
Appearance	Bloody	Clear
Red blood cell (cmm)	11,000	0-2,000
White blood cell (cmm)	5,86,160	0-20
Segmented cells (%)	97	0 -50
Lymphocytes (%)	2	0-50
Monocytes (%)	1	0 -50
Gram stain	Few polymorphonuclear leukocytes and many gram-positive cocci	Negative
Culture and sensitivity	Methicillin-sensitive *Staphylococcus aureus *&* Streptococcus mitis* group	Negative
Fungal and anaerobic culture	Negative	Negative
Acid-fast Bacilli	Negative	Negative
Crystal analysis	Negative	Negative

**Figure 3:**
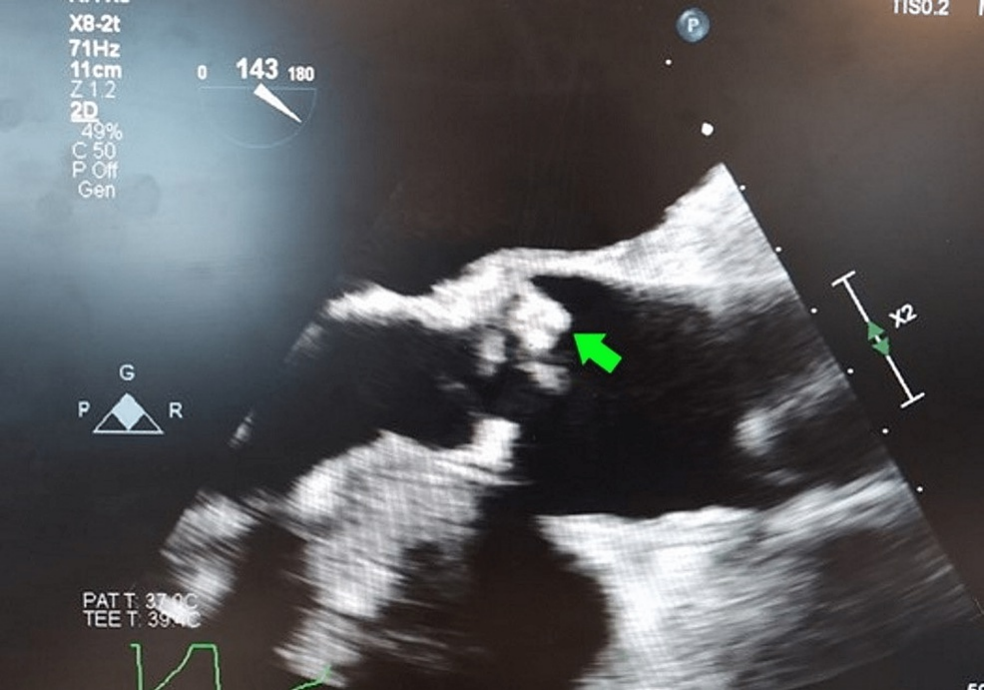
Transesophageal echocardiogram showing an aortic mass

One out of two blood culture bottles also grew MSSA, and the subsequent blood culture was negative. Transthoracic echocardiogram (TTE) was technically difficult due to his body habitus, but transesophageal echocardiogram (TEE) revealed a small mass measuring 0.8cm in its largest diameter on the aortic side of the right coronary cusp, concerning for valvular vegetations (Figure 3).

The patient was taken to the OR for incision and drainage of right knee effusion with wound vacuum-assisted closure (VAC) placement. His large prepatellar infected hematoma was drained with residual lateral and medial pockets, and necrotic tissue was debrided. A culture of necrotic tissue grew MSSA and *S. mitis*. He was started on IV cefazolin which was switched to IV daptomycin at discharge by an infectious disease specialist to enable once-daily antibiotic administration for a total of six weeks. His follow-up TTE was done after the completion of antibiotics and revealed no obvious vegetation within the limits of the study. He completed six weeks of antibiotics and regained the full function of his knee with physical therapy.

## Discussion

Septic prepatellar bursitis constitutes one-third of the total cases of prepatellar bursitis [3]. *Staphylococcus aureus* is the causative organism in 80% of the cases. In 55% of the cases, the predisposing factor is an overlying skin lesion related to occupational or recreational trauma. Immunosuppressive states like steroid use and diabetes mellitus also increase the risk of septic bursitis [4]. Typically, it presents as erythema, pain, swelling, and warmth of the prepatellar area. Careful examination is essential as the limitation of joint movement should raise suspicion for concurrent septic arthritis. Initial blood work shows leukocytosis with neutrophilia and elevation of sedimentation rate and CRP.

In diagnosing septic bursitis, bursa fluid aspirate should be sent for cell count, cytology, crystal analysis, gram stain, and culture. Bursa fluid count greater than 2000 cells/microliter and positive gram stain is consistent with septic bursitis. The absence of crystals rules out crystal-associated bursitis. In terms of imaging, plain radiographs should show soft tissue swelling with bursa fluid accumulation and synovial thickening. Two sets of blood cultures should be sent before initiating antibiotics. One-third of the cases of bursitis are associated with bacteremia, with *S. aureus* being the most common pathogen [5].

Our report features a case of a bricklayer with a history of type 2 diabetes mellitus and recurrent prepatellar bursitis, with prior catheter drainage of hemorrhagic bursa effusion. Bursa fluid aspiration revealed markedly elevated leukocyte count and grew MSSA and *S. mitis*, confirming septic bursitis. One out of two of the first sets of blood cultures grew *S. aureus*. Repeat blood cultures were negative.

Transthoracic echocardiography is recommended in Staphylococcal bacteremia to rule out IE. The Infectious Disease Society of America guidelines for methicillin-resistant *S. aureus *(MRSA) bacteremia recommends echocardiography for all patients with *S. aureus* bacteremia. The TEE is preferred as it is a more sensitive modality. It is important as the presence of concomitants impacts the decision on the duration of antimicrobial therapy [6]. This patient’s TTE revealed moderate aortic stenosis due to calcification but no valvular vegetations, however, a negative TTE is insufficient for ruling out IE because of its low sensitivity compared to TEE. We proceeded with performing TEE as the patient's preexisting degenerative aortic valve disease increased his risk of IE [7]. The TEE showed a calcified tri-leaflet aortic valve with a small mass attached to the aortic side of the right coronary cusp. These findings were consistent with endocarditis. Based on our literature review, there have been some case reports of IE complicated with septic bursitis and other rheumatic complications [8-10]. However, there are very few case reports on septic bursitis leading to IE [11]. The temporal event in this patient’s case suggests that his septic bursitis was complicated by endocarditis and not vice versa. His history of recurrent prepatellar bursitis related to overuse injury and recent catheter drainage of the hemorrhagic effusion predisposed him to septic bursitis and sepsis, while his underlying degenerative aortic valve disease was his risk factor for IE. The TTE is not indicated for gram-negative bacteremia unless risk factors like a prosthetic heart valve, injection drug abuse, and previous history of IE are present.

## Conclusions

Infective endocarditis is rarely secondary to septic prepatellar bursitis, but all patients with gram-positive bacteremia should have at least a TTE to rule out IE and be on the appropriate anti-staphylococcal antibiotics. Patients with underlying risk factors for IE are more predisposed, and appropriate patients with gram-negative bacteremia should also be considered for a work-up of IE. This case highlights the role of TEE in detecting IE in patients with an otherwise normal TTE.
